# Comparative hospitalization risk for SARS‐CoV‐2 Omicron and Delta variant infections, by variant predominance periods and patient‐level sequencing results, New York City, August 2021–January 2022

**DOI:** 10.1111/irv.13062

**Published:** 2022-10-31

**Authors:** Sharon K. Greene, Alison Levin‐Rector, Nang T. T. Kyaw, Elizabeth Luoma, Helly Amin, Emily McGibbon, Robert W. Mathes, Shama D. Ahuja

**Affiliations:** ^1^ Surveillance and Epidemiology Section, COVID‐19 Emergency Response Team New York City Department of Health and Mental Hygiene Long Island City New York USA; ^2^ Epidemic Intelligence Service Centers for Disease Control and Prevention Atlanta Georgia USA; ^3^ Bureau of the Public Health Laboratory New York City Department of Health and Mental Hygiene New York New York USA

**Keywords:** COVID‐19, epidemiology, hospitalization, informatics, public health, SARS‐CoV‐2, selection bias, whole genome sequencing

## Abstract

**Background:**

Comparing disease severity between SARS‐CoV‐2 variants among populations with varied vaccination and infection histories can help characterize emerging variants and support healthcare system preparedness.

**Methods:**

We compared COVID‐19 hospitalization risk among New York City residents with positive laboratory‐based SARS‐CoV‐2 tests when ≥98% of sequencing results were Delta (August–November 2021) or Omicron (BA.1 and sublineages, January 2022). A secondary analysis defined variant exposure using patient‐level sequencing results during July 2021–January 2022, comprising 1–18% of weekly confirmed cases.

**Results:**

Hospitalization risk was lower among patients testing positive when Omicron (16,025/488,053, 3.3%) than when Delta predominated (8268/158,799, 5.2%). In multivariable analysis adjusting for demographic characteristics and prior diagnosis and vaccination status, patients testing positive when Omicron predominated, compared with Delta, had 0.72 (95% CI: 0.63, 0.82) times the hospitalization risk. In a secondary analysis of patients with sequencing results, hospitalization risk was similar among patients infected with Omicron (2042/29,866, 6.8%), compared with Delta (1780/25,272, 7.0%), and higher among the subset who received two mRNA vaccine doses (adjusted relative risk 1.64; 95% CI: 1.44, 1.87).

**Conclusions:**

Hospitalization risk was lower among patients testing positive when Omicron predominated, compared with Delta. This finding persisted after adjusting for prior diagnosis and vaccination status, suggesting intrinsic virologic properties, not population‐based immunity, explained the lower severity. Secondary analyses demonstrated collider bias from the sequencing sampling frame changing over time in ways associated with disease severity. Representative data collection is necessary to avoid bias when comparing disease severity between previously dominant and newly emerging variants.

## 1 INTRODUCTION

Omicron (B.1.1.529 and BA lineages), a SARS‐CoV‐2 variant of concern, has caused less severe disease than prior variants in South Africa,^1^, ^2^ Europe,^3^, ^4^, ^5^, ^6^, ^7^, ^8^ and Canada.^9^ Similar findings have been reported in the United States, based on national surveillance data and a large healthcare database,^10^ healthcare systems,^11^, ^12^, ^13^, ^14^ and hospitals.^15^, ^16^ A limited number of studies to assess Omicron severity used population‐based surveillance data linked to immunization registry data in the United States^17^, ^18^ or used patient‐level whole‐genome sequencing (WGS) results.^12^, ^13^, ^18^, ^19^

Diminished disease severity could result from Omicron's intrinsic virologic properties (e.g., lower replication competence in human lungs^20^), its emergence among populations with vaccination‐ and infection‐induced immunity, or a combination thereof. Assessing variant severity in populations with varied vaccination and infection histories can help to clarify reasons for lower disease severity and support healthcare system preparedness.

In New York City (NYC), Delta (B.1.617.2 and AY lineages) became predominant (i.e., >50% of sequenced specimens) the week ending July 3, 2021; Omicron became predominant the week ending December 18, 2021.^21^ Delta constituted 96% of sequencing results the first week of December 2021 and was swiftly displaced by Omicron, with 92% of sequencing results by the last week of December 2021.^21^ By the time Omicron was introduced into NYC, substantial percentages of the population had been previously infected (a quarter of NYC residents were estimated to have been infected by mid‐2020 before vaccine availability)^22^, ^23^ and vaccinated (72% completed the primary vaccine series and 21% had received an additional dose as of December 18, 2021).^24^ During December 12, 2021–January 29, 2022, a total of 1,047,428 confirmed and probable cases of COVID‐19 were diagnosed among NYC residents.^25^ Given this surge in infections, we sought to leverage population‐based surveillance, WGS, and immunization registry data to compare disease severity between Omicron and Delta infections, accounting for prior vaccination and diagnosis history.

## 2 METHODS

### 2.1 Infection with Delta and Omicron variants

Clinical and commercial laboratories are required to report SARS‐CoV‐2 test results for New York State residents through the New York State Electronic Clinical Laboratory Reporting System (ECLRS).^26^ For some SARS‐CoV‐2 infections diagnosed among NYC residents, WGS is performed by the NYC Department of Health and Mental Hygiene (DOHMH) Public Health Laboratory, the Pandemic Response Laboratory,^27^ and other laboratories reporting through ECLRS.^28^ The Pandemic Response Laboratory reported most sequencing results during the study period, including 85% of sequencing results among patients tested during January 2022. Variant assignment for sequence data from the Pandemic Response Laboratory and DOHMH were analyzed by the Public Health Laboratory with Pangolin version 4.0.6 and data from other laboratories with Pangolin versions 3.1.8–3.1.20.

With the use of a cohort study design, we defined a cohort of patients presumed infected with Delta as testing positive by laboratory‐based SARS‐CoV‐2 molecular or antigen testing during August–November 2021 (≥98% of sequencing results were Delta during each week ending August 7–November 27, 2021).^21^ We defined a cohort of patients presumed infected with Omicron as testing positive during January 2022 (≥99% of sequencing results were Omicron during each week ending January 8–29, 2022).^21^ Diagnosis date was defined as the specimen collection date of the first positive test within a 90‐day period.

In a secondary analysis, we restricted to patients with sequencing results to eliminate uncertainty in the variant causing infection, at the expense of reduced sample size and representativeness. We identified patients testing positive for SARS‐CoV‐2 infection during July 1, 2021–January 31, 2022 and with sequencing results indicating infection with Delta (N = 25,272) or Omicron (N = 29,866) variant. The weekly percentage of confirmed cases with sequencing results ranged from a high of 18% of patients with specimens collected during week ending December 4, 2021 to a low of 1% during week ending January 1, 2022.^29^ At the Omicron wave's peak, the Pandemic Response Laboratory and Public Health Laboratory prioritized specimens with low cycle threshold values for sequencing.

**FIGURE 1:**
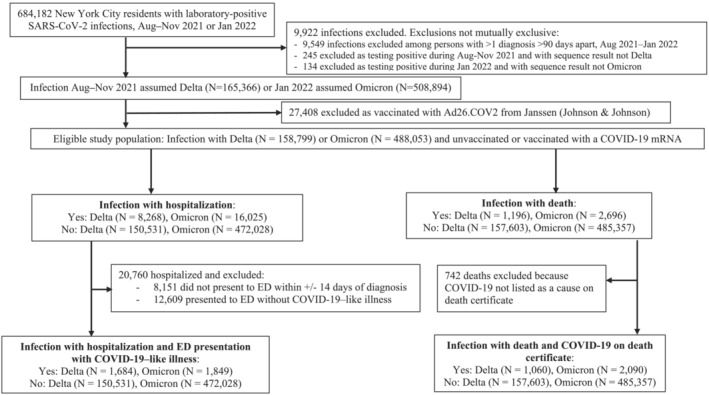
Eligibility for analysis of New York City residents testing positive for SARS‐CoV‐2 infection during periods of Omicron or Delta variant predominance.

Exclusion criteria included patients with >1 diagnosis >90 days apart during the study period, such that each eligible patient had only Delta or Omicron infection (Figure 1). Additionally, on December 16, 2021, the Advisory Committee on Immunization Practices preferentially recommended the mRNA COVID‐19 vaccines.^30^ The limited percentage of study participants whose first COVID‐19 vaccine dose was Ad26.COV2 from Janssen (Johnson & Johnson) were excluded (4% [27,408/674,260], Figure 1). With the operational benefit of requiring only one dose, this vaccine had been differentially administered to certain populations, for example, homebound seniors.^31^ These vaccine recipients likely disproportionately had underlying conditions associated with COVID‐19 hospitalization and death; including them without accounting for these unobserved confounders could have biased results.

### 2.2 Hospitalization and death

COVID‐19 hospitalizations and deaths are ascertained by importing and matching data from supplemental systems.^32^ Supplemental hospitalization data were obtained from emergency department syndromic surveillance, regional health information organizations, public hospitals, DOHMH's electronic death registry system, and hospitals' electronic health record systems.^32^ These systems do not capture all hospitalizations, and although all systems capture fact of hospitalization, the underlying cause is not necessarily available. Thus, people hospitalized because of COVID‐19 illness could not be distinguished from people hospitalized because SARS‐CoV‐2 infection exacerbated an underlying condition or because SARS‐CoV‐2 infection was an incidental diagnosis.^33^ Incidental diagnoses were likely disproportionately common while Omicron predominated, given high infection prevalence.^34^

COVID‐19 hospitalizations were defined as NYC residents whose positive SARS‐CoV‐2 test was within 14 days before or 3 days after hospital admission. COVID‐19 deaths were defined as NYC residents with a positive SARS‐CoV‐2 test, and (a) the cause‐of‐death on the death certificate was COVID‐19 or similar; or (b) COVID‐19 was not a cause‐of‐death on the death certificate but the patient died within 30 days of COVID‐19 diagnosis, and the death was not attributable to external causes, such as injury.^32^

Sensitivity analyses applied more specific definitions. For a more specific hospitalization definition, we restricted to the subset of COVID‐19 hospitalizations for which, based on syndromic surveillance, patients had presented to the emergency department with COVID‐19‐like illness within +/− 14 days of diagnosis. COVID‐19‐like illness was defined as chief complaint or ICD‐10 codes for pneumonia or influenza‐like illness (i.e., fever, cough, sore throat, and/or respiratory illness) and without regard to the COVID‐19 ICD‐10 code. For a more specific deaths definition, we restricted to the subset with COVID‐19 indicated on the death certificate.

### 2.3 Vaccination status

By matching with NYC's Citywide Immunization Registry, patients were assigned a vaccination status indicating the number of valid recorded doses (0–3) of an mRNA vaccine (BNT162b2 from Pfizer‐BioNTech or mRNA‐1273 from Moderna) received ≥14 days before COVID‐19 diagnosis.^35^ We restricted third doses to those administered starting August 13, 2021, when the Advisory Committee on Immunization Practices recommended an additional dose after an initial series for eligible immunocompromised persons.^36^ Doses administered outside of New York State and by federal entities might have been missed, reducing the number of doses ascertained per patient.

### 2.4 Diagnosis history

Prior COVID‐19 diagnosis was defined as a positive laboratory‐based molecular or antigen test >90 days before diagnosis with Delta or Omicron infection. Repeat positive tests are considered possible reinfections after 90 days, per the surveillance case definition.^37^ Prior diagnoses would have been missed among patients who resided outside of NYC when tested previously or who did not access testing, for instance during spring 2020 when testing availability was limited.^32^

### 2.5 Statistical analysis

The exposure of interest was infection with the Omicron or Delta variant, and the outcomes were hospitalization or death. Crude and adjusted relative risks (aRRs) and 95% confidence intervals (CIs) were calculated using Poisson regression with robust error variance.^38^ Models adjusted for gender, age group (for hospitalizations: in 10‐year groupings as in Bager et al.^3^: <10, 10–19, …, 80–89, ≥90 years; for deaths: the same, except aggregating those aged <30 years), congregate setting residence (nursing home, jail, or prison; yes/no), and for community‐dwelling residents, neighborhood poverty level (percent of residents based on census tract as of diagnosis with incomes below the federal poverty level, per the American Community Survey, 2015–2019).

To account for infection‐induced immunity, we adjusted for prior COVID‐19 diagnosis (yes/no) and, if yes, number of days since most recent prior diagnosis (91–179, 180–269, 270–359, 360–449, ≥450 days). Similarly, to account for vaccine‐induced immunity, we adjusted for number of mRNA vaccine doses received ≥14 days before COVID‐19 diagnosis (0–1, 2, or 3 doses) and number of days since the 14‐day period had passed after the most recent dose (<90, 90–179, 180–269, ≥270 days). Patients with 0 and 1 doses were aggregated for comparability with similar studies^3^, ^13^ and because a limited number of patients had received only 1 dose of the 2‐dose primary series.

We included an interaction term for variant (Omicron vs. Delta) and vaccination status (0–1, 2, or 3 doses) and assessed risk for poor outcomes among patients with Omicron infection compared with Delta infection within each vaccination status stratum. As in Bager et al.,^3^ we assessed risk for poor outcomes for both Omicron and Delta infections by vaccination status, compared with patients with Delta infection and 0–1 mRNA vaccine doses received ≥14 days before diagnosis. Finally, restricting to patients with Omicron infection, we assessed risk for poor outcomes by vaccination status, compared with patients with 0–1 mRNA vaccine dose received ≥14 days before COVID‐19 diagnosis.

Data were extracted from the DOHMH COVID‐19 surveillance database (Maven® Disease Surveillance and Outbreak Management System; Conduent, Florham Park, New Jersey), on March 10, 2022. Analyses were conducted using SAS® Enterprise Guide, version 7.1 (SAS Institute, Inc., Cary, North Carolina). This activity was deemed public health surveillance that is nonresearch by the DOHMH Institutional Review Board.

## 3 RESULTS

### 3.1 Descriptive analysis

After applying study exclusion criteria, the eligible population included 158 799 NYC residents with a positive laboratory‐based SARS‐CoV‐2 test during August–November 2021, when Delta predominated, in addition to 488 053 persons testing positive during January 2022, when Omicron predominated (Figure 1, Table 1). Of 21,023 persons testing positive during August–November 2021 and with a Delta sequencing result, lineages constituting ≥5% of sequencing results were AY.103 (17%), B.1.617.2 (14%), AY.3 (10%), AY.44 (9%), AY.25 (7%), and AY.25.1 (6%). Of 19,274 persons testing positive during January 2022 and with an Omicron sequencing result, lineages constituting ≥5% of sequencing results were BA.1.1 (42%), BA.1 (39%), BA.1.15 (6%), and BA.1.17.2 (6%); BA.1 and sublineages constituted 95.5% of sequencing results, with BA.2 and sublineages constituting the remaining 0.5%.Demographic characteristics were similar among cohorts; patients were predominantly female, aged 20–39 years, and residents of medium poverty areas (Table 1). Patients testing positive during Omicron predominance were more likely to have been vaccinated with 1, 2, and 3 doses than patients testing positive during Delta predominance (Table 1), which was expected because the Omicron wave occurred after the Delta wave, allowing more time for vaccine administration. Because limited temporal overlap was observed between the Delta wave and third vaccine dose administrations,^24^ only 0.3% of patients testing positive during Delta predominance had received 3 doses (Table 1).

**TABLE 1:** Characteristics of New York City residents who tested positive for SARS‐CoV‐2 infection during periods of Omicron (January 2022) and Delta (August–November 2021) variant predominance

	Diagnosis of SARS‐CoV‐2 infection		Hospitalized		Died	
	Omicron	Delta	Omicron	Delta	Omicron	Delta
	N (%)	N (%)	N (%)	N (%)	N (%)	N (%)
Total	488,053	158,799	16,025	8268	2696	1196
Gender						
Female	268,378 (55.0%)	83,542 (52.6%)	8492 (53.0%)	4249 (51.4%)	1255 (46.6%)	561 (46.9%)
Male	216,505 (44.4%)	74,837 (47.1%)	7526 (47.0%)	4017 (48.6%)	1441 (53.4%)	635 (53.1%)
Unknown or missing	3170 (0.6%)	420 (0.3%)	7 (0.0%)	2 (0.0%)	0 (0.0%)	0 (0.0%)
Age group (years)						
<10	61,075 (12.5%)	17,007 (10.7%)	737 (4.6%)	277 (3.4%)	2 (0.1%)	0 (0.0%)
10–19	65,319 (13.4%)	18,946 (11.9%)	423 (2.6%)	219 (2.6%)	1 (0.0%)	1 (0.1%)
20–29	75,672 (15.5%)	34,207 (21.5%)	1141 (7.1%)	690 (8.3%)	11 (0.4%)	5 (0.4%)
30–39	81,844 (16.8%)	33,104 (20.8%)	1501 (9.4%)	1060 (12.8%)	42 (1.6%)	32 (2.7%)
40–49	66,242 (13.6%)	19,879 (12.5%)	1246 (7.8%)	928 (11.2%)	84 (3.1%)	67 (5.6%)
50–59	60,271 (12.3%)	15,384 (9.7%)	1965 (12.3%)	1271 (15.4%)	176 (6.5%)	159 (13.3%)
60–69	43,038 (8.8%)	11,154 (7.0%)	2640 (16.5%)	1407 (17.0%)	432 (16.0%)	222 (18.6%)
70–79	21,743 (4.5%)	5952 (3.7%)	2801 (17.5%)	1235 (14.9%)	650 (24.1%)	309 (25.8%)
80–89	9546 (2.0%)	2447 (1.5%)	2465 (15.4%)	851 (10.3%)	746 (27.7%)	235 (19.6%)
≥90	3165 (0.6%)	688 (0.4%)	1106 (6.9%)	330 (4.0%)	552 (20.5%)	166 (13.9%)
Unknown or missing	138 (0.0%)	31 (0.0%)	0 (0.0%)	0 (0.0%)	0 (0.0%)	0 (0.0%)
Race/ethnicity						
Non‐Hispanic Asian/Pacific Islander	67,981 (13.9%)	12,919 (8.1%)	1236 (7.7%)	510 (6.2%)	267 (9.9%)	83 (6.9%)
Non‐Hispanic Black/African American	64,382 (13.2%)	26,961 (17.0%)	4624 (28.9%)	2436 (29.5%)	769 (28.5%)	329 (27.5%)
Hispanic/Latino	128,899 (26.4%)	33,446 (21.1%)	4592 (28.7%)	2253 (27.2%)	604 (22.4%)	267 (22.3%)
Non‐Hispanic White	98,484 (20.2%)	51,385 (32.4%)	4169 (26.0%)	2288 (27.7%)	924 (34.3%)	425 (35.5%)
Non‐Hispanic Other	3139 (0.6%)	2940 (1.9%)	202 (1.3%)	250 (3.0%)	68 (2.5%)	55 (4.6%)
Unknown	125,168 (25.6%)	31,148 (19.6%)	1202 (7.5%)	531 (6.4%)	64 (2.4%)	37 (3.1%)
Congregate setting resident^a^						
Yes	5447 (1.1%)	1144 (0.7%)	883 (5.5%)	326 (3.9%)	476 (17.7%)	150 (12.5%)
No	458,578 (94.0%)	156,365 (98.5%)	14,671 (91.6%)	7895 (95.5%)	2183 (81.0%)	1046 (87.5%)
Unknown or missing	24,028 (4.9%)	1290 (0.8%)	471 (2.9%)	47 (0.6%)	37 (1.4%)	0 (0.0%)
Census tract‐based poverty level^b^						
Low	133,738 (27.4%)	52,927 (33.3%)	3514 (21.9%)	2154 (26.1%)	701 (26.0%)	369 (30.9%)
Medium	150,231 (30.8%)	48,374 (30.5%)	4673 (29.2%)	2451 (29.6%)	908 (33.7%)	366 (30.6%)
High	90,218 (18.5%)	27,329 (17.2%)	3333 (20.8%)	1675 (20.3%)	508 (18.8%)	217 (18.1%)
Very high	84,124 (17.2%)	25,877 (16.3%)	3728 (23.3%)	1776 (21.5%)	491 (18.2%)	217 (18.1%)
Unknown or missing	29,742 (6.1%)	4292 (2.7%)	777 (4.8%)	212 (2.6%)	88 (3.3%)	27 (2.3%)
Prior COVID‐19 diagnosis						
Yes	42,115 (8.6%)	6057 (3.8%)	1118 (7.0%)	282 (3.4%)	106 (3.9%)	41 (3.4%)
No	445,938 (91.4%)	152,742 (96.2%)	14,907 (93.0%)	7986 (96.6%)	2590 (96.1%)	1155 (96.6%)
Days since prior diagnosis						
91–180	1052 (2.5%)	776 (12.8%)	23 (2.1%)	72 (25.5%)	2 (1.9%)	16 (39.0%)
180–269	2485 (5.9%)	1762 (29.1%)	64 (5.7%)	115 (40.8%)	5 (4.7%)	15 (36.6%)
270–359	15,581 (37.0%)	1211 (20.0%)	369 (33.0%)	35 (12.4%)	37 (34.9%)	2 (4.9%)
360–449	12,268 (29.1%)	1326 (21.9%)	281 (25.1%)	18 (6.4%)	20 (18.9%)	2 (4.9%)
≥450	10,729 (25.5%)	972 (16.0%)	381 (34.1%)	42 (14.9%)	42 (39.6%)	6 (14.6%)
Number of COVID‐19 vaccine doses^c^						
0	190,611 (39.1%)	102,939 (64.8%)	7935 (49.5%)	6139 (74.3%)	1413 (52.4%)	850 (71.1%)
1	27,574 (5.6%)	4417 (2.8%)	902 (5.6%)	252 (3.0%)	121 (4.5%)	40 (3.3%)
2	200,284 (41.0%)	50,897 (32.1%)	5558 (34.7%)	1849 (22.4%)	865 (32.1%)	301 (25.2%)
3	69,584 (14.3%)	546 (0.3%)	1630 (10.2%)	28 (0.3%)	297 (11.0%)	5 (0.4%)
Days since 14‐day period had passed after most recent COVID‐19 vaccine dose						
<90	116,427 (39.1%)	9922 (17.8%)	2498 (30.9%)	422 (19.8%)	373 (29.1%)	55 (15.9%)
90–179	55,478 (18.7%)	27,350 (49.0%)	1572 (19.4%)	961 (45.1%)	213 (16.6%)	159 (46.0%)
180–269	94,678 (31.8%)	17,630 (31.6%)	2544 (31.4%)	697 (32.7%)	401 (31.3%)	127 (36.7%)
≥270	28,868 (9.7%)	741 (1.3%)	1437 (17.8%)	34 (1.6%)	296 (23.1%)	4 (1.2%)

^a^ Congregate settings defined as having a residential address as of diagnosis of a nursing home, jail, or prison.

^b^ Low poverty defined as <10% of residents below the federal poverty level, medium as 10% to <20%, high as 20 to <30%, and very high as ≥30%.

^c^ A second dose of the BNT162b2 vaccine was considered valid if it was administered ≥17 days (3 weeks minus 4‐day grace period) after a first dose of BNT162b2 or ≥28 days after a first dose of mRNA‐1273. A second dose of the mRNA‐1273 vaccine was considered valid if it was administered ≥24 days (4 weeks minus a 4‐day grace period) after a first dose of mRNA‐1273 or ≥28 days after a first dose of BNT162b2. A third dose was considered valid if it was administered ≥150 days after a second dose of vaccine from either manufacturer.

History of prior COVID‐19 diagnosis was more common among patients testing positive during Omicron (8.6%) than Delta predominance (3.8%) (Table 1). Study eligibility required patients to have had either Delta or Omicron infection but not both. Thus, with Omicron emerging after Delta, patients infected with Omicron had on average more time for infection‐induced immunity to wane. The median number of days since prior diagnosis for Omicron reinfections was 367 (interquartile range: 317–467) and for Delta reinfections was 300 (interquartile range: 224–365).

Missingness for covariates included in regression analyses was negligible (≤1.5%), except variables depending on geocoding (i.e., congregate setting residence and neighborhood poverty level) had up to 6.1% missingness (Table 1). We conducted regression modeling using complete case analysis, assuming data were missing completely at random.

### 3.2 Hospitalization and death

**FIGURE 2:**
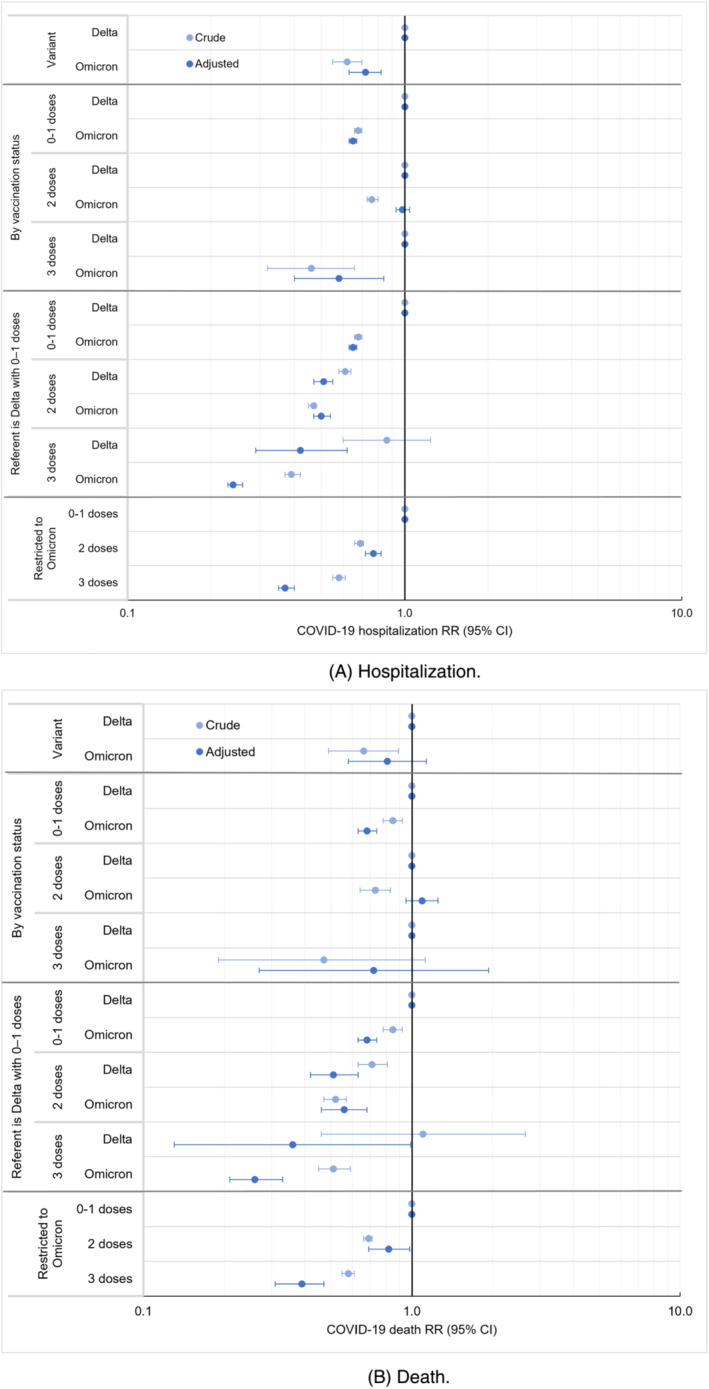
Relative risk (RR) for COVID‐19 hospitalization and death among patients testing positive during periods of Omicron compared with Delta variant predominance, overall and according to vaccination status, New York City, August 2021–January 2022. (A) Hospitalization. (B) Death.

Of 488,053 NYC residents testing positive during weeks when ≥99% of sequencing results were Omicron and presumed infected with Omicron, 16,025 (3.3%) were hospitalized, and 2696 (0.6%) died. Of 158,799 persons testing positive when ≥98% of sequencing results were Delta and presumed infected with Delta, 8268 (5.2%) were hospitalized, and 1196 (0.8%) died. Patients infected with Omicron compared with Delta had 0.72 (95% CI: 0.63, 0.82) times the hospitalization risk, adjusting for gender, age, congregate setting residence, neighborhood poverty level, prior COVID‐19 diagnosis, time since prior COVID‐19 diagnosis, number of mRNA vaccine doses received, and number of days since the 14‐day period had passed after the most recent vaccine dose (Figure 2, Table S2). The point estimate for deaths among patients infected with Omicron compared with Delta was similar to that of hospitalizations but with wider uncertainty (aRR 0.81; 95% CI: 0.58, 1.13) (Figure 2, Table S3). In sensitivity analyses using more specific outcome definitions with sparser observations (Figure 1), point estimates remained similar for severity of Omicron compared with Delta infection, with 0.70 (95% CI: 0.36, 1.34) times the adjusted risk for hospitalization with COVID‐19‐like illness presentation and 0.86 (95% CI: 0.54, 1.38) times the adjusted risk for death with COVID‐19 indicated on the death certificate (Figure S2).

Associations between poor outcomes and variant exposure were modified by vaccination status; the interaction term for variant and vaccination status in adjusted models was *p* < 0.0001 for all four outcomes (hospitalization, hospitalization with COVID‐19‐like illness presentation, death, and death with COVID‐19 indicated on the death certificate). By vaccination status, aRR for hospitalization among patients testing positive during Omicron predominance, compared with Delta, was 0.65 (95% CI: 0.63, 0.67) among patients with 0–1 vaccine dose, 0.98 (95% CI: 0.93, 1.04) among those who received 2 doses, and 0.58 (95% CI: 0.40, 0.84) among those who received 3 doses (Figure 2, Table S2). The pattern for all four outcomes was similar, in that severity among patients testing positive during Omicron predominance, compared with Delta, was strongly reduced among patients who received 0–1 vaccine dose but less reduced or not reduced among those who received 2 doses (Figure 2, Figure S2). Comparative severity estimates among patients who received 3 doses had wide uncertainty given the limited number of patients who both tested positive when Delta predominated and had received 3 doses. However, restricting to patients testing positive during Omicron predominance and compared with those with 0–1 dose, those who received 2 vaccine doses had 0.77 times the adjusted hospitalization risk (95% CI: 0.72, 0.82) and 0.82 times the adjusted death risk (95% CI: 0.69, 0.98); those who received 3 doses had 0.37 times the adjusted hospitalization risk (95% CI: 0.35, 0.40) and 0.39 times the adjusted death risk (95% CI: 0.31, 0.47) (Figure 2, Tables S2 and S3).

Furthermore, we assessed risk for poor outcomes by vaccination status, compared with patients testing positive during Delta predominance who received 0–1 vaccine dose. Risk for hospitalization after 2 doses was similar between patients with Omicron (aRR 0.50; 95% CI: 0.47, 0.54) and Delta infections (aRR 0.51; 95% CI: 0.47, 0.55), and hospitalization risk after 3 vaccine doses was lower with Omicron (aRR 0.24; 95% CI: 0.23, 0.26) than with Delta (aRR 0.42; 95% CI: 0.29, 0.62) (Table S2).

Results were not robust in secondary analyses restricting to patients with sequencing results (Figure S1). Of 29 866 NYC residents with Omicron sequencing results, 2042 (6.8%) were hospitalized and 446 (1.5%) died (Table S1). Of 25,272 patients with Delta sequencing results, 1780 (7.0%) were hospitalized, and 331 (1.3%) died. The aRR for hospitalization among patients who, based on sequencing results, were infected with Omicron, compared with Delta, was increased among those who received 2 doses (aRR 1.64; 95% CI: 1.44, 1.87) (Figure S3). This finding of increased comparative severity for Omicron infections was inconsistent with both the primary analysis and prior literature and likely reflects a bias in which specimens from severely ill patients were disproportionately selected for sequencing when Omicron predominated.

## 4 DISCUSSION

NYC residents testing positive for SARS‐CoV‐2 infection when Omicron (BA.1 and sublineages) predominated, compared with when Delta predominated, had lower hospitalization risk. This finding was consistent in both crude and adjusted analyses controlling for prior diagnosis and vaccination status, and we found lower hospitalization and death risk among patients with 0–1 vaccine dose, suggesting Omicron's diminished disease severity is likely more attributable to intrinsic virologic properties than to prior population‐based immunity. Among patients with 2 vaccine doses, those infected with Omicron had similar hospitalization and death risk as those infected with Delta, possibly reflecting Omicron's increased ability to evade vaccine‐induced immunity.^39^, ^40^ Analyses did not include persons only testing positive using at‐home rapid antigen tests, which became more widely available by mid‐December 2021 when Omicron predominated.^41^, ^42^ If at‐home tests were used differentially by persons with milder illness, then patients testing positive while Omicron predominated would have been biased toward severe illness. Thus, our estimates are likely conservative, and Omicron could be even less severe, compared with Delta, than we report. Despite reduced comparative severity, the absolute numbers of COVID‐19 hospitalizations and deaths were higher when Omicron predominated given the volume of Omicron infections.

We analyzed 646,852 persons testing positive when Delta or Omicron predominated, >3 times as large a study population as the 188,980 persons included in a similar, Danish analysis.^3^ Findings were consistent, except the Danish analysis showed that among those who received 2 vaccine doses, patients infected with Omicron compared with Delta had lower hospitalization risk (aRR 0.71; 95% CI: 0.60, 0.86), whereas our study showed no difference (aRR 0.98; 95% CI: 0.93, 1.04). Reasons for this discrepancy are unclear, although the confounder adjustment set was different (we adjusted for congregate setting residence, neighborhood poverty level, time since vaccination, and time since prior COVID‐19 diagnosis; whereas the Danish analysis adjusted for presence of comorbidities, which was unavailable for our cohort), and vaccination information could have been more complete from the Danish national registry.

In addition to the large population, other study strengths included an assessment of multiple poor COVID‐19 outcomes (hospitalization, hospitalization with COVID‐19‐like illness presentation, death, and death with COVID‐19 indicated on the death certificate). The consistency of findings across sensitive and specific outcome definitions suggested that bias in outcome ascertainment was minimal, despite the higher infection prevalence when Omicron predominated. In addition, we demonstrated using real‐world surveillance data the potential limitations of restricting to patients with WGS results to assess poor outcomes. A secondary analysis implausibly indicated higher disease severity among patients with Omicron sequencing results, compared with patients with Delta sequencing results, among those who received 2 vaccine doses. Conditioning analyses on patients seeking voluntary testing and having specimens selected for sequencing, the probabilities of which changed over time concurrent with changing variant predominance and temporary protocols to prioritize specimens with low cycle threshold values for sequencing, likely induced collider bias.^43^, ^44^ This underscores the importance of a representative WGS sampling frame that does not change over time in ways associated with disease severity. Outcome comparisons among patients with sequencing results should be interpreted cautiously if not diagnosed closely in time. Sample size calculations for variant surveillance should consider not only how many sequences are needed to detect new variants or to monitor variant prevalence^45^ but also the much larger number needed to compare risk for hospitalization and death across variants, stratifying by vaccination status, to support healthcare capacity planning.

SARS‐CoV‐2 variant surveillance exemplifies investments in public health data and informatics. Our large, population‐based study relied on DOHMH capacity to link confidential, identifiable patient data in the COVID‐19 disease registry with laboratory data for sequencing results, with the immunization registry for vaccination status, with past cases for diagnosis history, with supplemental data sources for hospitalizations, and with the vital statistics registry for deaths. Capacity to access and link data sources varies across U.S. health departments.^46^ Although we did not have access to all relevant variables (e.g., comorbidities, therapeutics receipt, intensive care unit admission, and mechanical ventilation), such data are more readily available within healthcare systems.^11^ Strengthening linkages between healthcare and public health data systems and collecting data representatively are necessary to avoid bias when comparing disease severity between previously dominant and newly emerging SARS‐CoV‐2 variants and subvariants (e.g., BA.5). Conducting such comparisons, while accounting for waning immunity from prior infections and vaccinations, is important for anticipating demands on the healthcare system. Among NYC residents who tested positive when Omicron predominated and compared with those with 0–1 mRNA vaccine dose, those who received 3 doses had strongly reduced risk for hospitalization and death, supporting continued efforts to ensure up‐to‐date vaccination coverage.

## CONFLICT OF INTEREST

None.

## DISCLAIMER

The findings and conclusions in this report are those of the authors and do not necessarily represent the official position of the New York City Department of Health and Mental Hygiene or the Centers for Disease Control and Prevention.

## AUTHOR CONTRIBUTIONS

Sharon K. Greene performed the conceptualization (lead), methodology (lead), supervision (supporting), writing—original draft (lead), and writing—review & editing (equal). Alison Levin‐Rector performed the data curation (lead), formal analysis (lead), investigation (equal), methodology (supporting), software (lead), visualization (lead), and writing—review & editing (equal). Nang T.T. Kyaw performed the formal analysis (supporting), software (supporting), validation (supporting), visualization (supporting), and writing—review & editing (equal). Elizabeth Luoma performed the data curation (equal), investigation (equal), project administration (equal), resources (equal), and writing—review & editing (equal). Helly Amin performed the data curation (equal), investigation (equal), resources (equal), and writing—review & editing (equal). Emily McGibbon performed the data curation (equal), formal analysis (supporting); investigation (equal), resources (equal), software (supporting), validation (lead), and writing—review & editing (equal). Robert W. Mathes performed the data curation (equal), investigation (equal), resources (equal), and writing—review & editing (equal). Shama D. Ahuja performed the project administration (equal), resources (equal), supervision (lead), and writing—review & editing (equal)**.**

## Supporting information


**Table S1.** Characteristics of New York City residents with SARS‐CoV‐2 Delta and Omicron variant infections based on sequencing result, July 2021–January 2022.
**Table S2**. Relative risk for COVID‐19 hospitalization among patients testing positive for SARS‐CoV‐2 infection during periods of Omicron compared with Delta predominance, overall and according to vaccination status, New York City, August 2021–January 2022.
**Table S3.** Relative risk for COVID‐19 death among patients testing positive for SARS‐CoV‐2 infection during periods of Omicron compared with Delta predominance, overall and according to vaccination status, New York City, August 2021–January 2022.
**Figure S1.** Eligibility for secondary analysis of New York City residents with SARS‐CoV‐2 Delta or Omicron variant infection based on whole‐genome sequencing result.
**Figure S2.** Relative risk (RR) for (A) hospitalization with COVID‐19–like illness presentation and (B) death with COVID‐19 indicated on the death certificate, among patients testing positive for SARS‐CoV‐2 infection during periods of Omicron compared with Delta predominance overall and according to vaccination status, New York City, August 2021–January 2022.
**Figure S3.** Relative risk (RR) for COVID‐19 hospitalization and death among patients with Omicron compared with Delta sequencing results, overall and according to vaccination status, New York City, July 2021–January 2022.

## Data Availability

Line‐level data are not publicly available in accordance with patient confidentiality and privacy laws. Publicly available data are available from https://www1.nyc.gov/site/doh/covid/covid-19-data.page.
