# Supplementary material for: Comparative hospitalization risk for SARS‐CoV‐2 Omicron and Delta variant infections, by variant predominance periods and patient‐level sequencing results, New York City, August 2021–January 2022
Source: Influenza Other Respir Viruses. 2022 Oct 31;17(1):e13062. doi: 10.1111/irv.13062 (PMC9835408; doi:10.1111/irv.13062)
Supplement: Supplementary file 1 — Table S1. Characteristics of New York City residents with SARS‐CoV‐2 Delta and Omicron variant infections based on sequencing result, July 2021–January 2022. Table S2. Relative risk for COVID‐19 hospitalization among patients testing positive for SARS‐CoV‐2 infection during periods of Omicron compared with Delta predominance, overall and according to vaccination status, New York City, August 2021–January 2022. Table S3. Relative risk for COVID‐19 death among patients testing positive for SARS‐CoV‐2 infection during periods of Omicron compared with Delta predominance, overall and according to vaccination status, New York City, August 2021–January 2022. Figure S1. Eligibility for secondary analysis of New York City residents with SARS‐CoV‐2 Delta or Omicron variant infection based on whole‐genome sequencing result. Figure S2. Relative risk (RR) for (A) hospitalization with COVID‐19–like illness presentation and (B) death with COVID‐19 indicated on the death certificate, among patients testing positive for SARS‐CoV‐2 infection during periods of Omicron compared with Delta predominance overall and according to vaccination status, New York City, August 2021–January 2022. Figure S3. Relative risk (RR) for COVID‐19 hospitalization and death among patients with Omicron compared with Delta sequencing results, overall and according to vaccination status, New York City, July 2021–January 2022. [file IRV-17-0-s001.doc]

**SUPPORTING INFORMATION**

Greene SK, Levin-Rector A, Kyaw NTT, et al. Comparative hospitalization risk for SARS-CoV-2 Omicron and Delta variant infections, by variant predominance periods and patient-level sequencing results, New York City, August 2021–January 2022.

**Table S1.** Characteristics of New York City residents with SARS-CoV-2 Delta and Omicron variant infections based on sequencing result, July 2021–January 2022.

**Table S2**. Relative risk for COVID-19 hospitalization among patients testing positive for SARS-CoV-2 infection during periods of Omicron compared with Delta predominance, overall and according to vaccination status, New York City, August 2021–January 2022.

**Table S3.** Relative risk for COVID-19 death among patients testing positive for SARS-CoV-2 infection during periods of Omicron compared with Delta predominance, overall and according to vaccination status, New York City, August 2021–January 2022.

**Figure S1.** Eligibility for secondary analysis of New York City residents with SARS-CoV-2 Delta or Omicron variant infection based on whole-genome sequencing result.

**Figure S2.** Relative risk (RR) for (A) hospitalization with COVID-19–like illness presentation and (B) death with COVID-19 indicated on the death certificate, among patients testing positive for SARS-CoV-2 infection during periods of Omicron compared with Delta predominance overall and according to vaccination status, New York City, August 2021–January 2022.

**Figure S3.** Relative risk (RR) for COVID-19 hospitalization and death among patients with Omicron compared with Delta sequencing results, overall and according to vaccination status, New York City, July 2021–January 2022.

**Table S1. Characteristics of New York City residents with SARS-CoV-2 Delta and Omicron variant infections based on sequencing result, July 2021–January 2022.**

|  | **Diagnosis of SARS-CoV-2 Infection** | | **Hospitalized** | | **Died** | |
| --- | --- | --- | --- | --- | --- | --- |
|  | Omicron N (%) | Delta  N (%) | Omicron N (%) | Delta N (%) | Omicron  N (%) | Delta N (%) |
| **Total** | 29,866 | 25,272 | 2,042 | 1,780 | 446 | 331 |
|  |  |  |  |  |  |  |
| **Gender** |  |  |  |  |  |  |
| **Female** | 16,182 (54.2%) | 13,273 (52.5%) | 1,065 (52.2%) | 925 (52.0%) | 181 (40.6%) | 152 (45.9%) |
| **Male** | 13,250 (44.4%) | 11,957 (47.3%) | 977 (47.8%) | 854 (48.0%) | 265 (59.4%) | 179 (54.1%) |
| **Unknown or missing** | 434 (1.5%) | 42 (0.2%) | 0 (0.0%) | 1 (0.1%) | 0 (0.0%) | 0 (0.0%) |
|  |  |  |  |  |  |  |
| **Age group (years)** |  |  |  |  |  |  |
| **<10** | 2,998 (10.0%) | 2,612 (10.3%) | 126 (6.2%) | 43 (2.4%) | 0 (0.0%) | 0 (0.0%) |
| **10–19** | 3,049 (10.2%) | 2,906 (11.5%) | 70 (3.4%) | 48 (2.7%) | 0 (0.0%) | 0 (0.0%) |
| **20–29** | 5,771 (19.3%) | 5,087 (20.1%) | 158 (7.7%) | 133 (7.5%) | 7 (1.6%) | 1 (0.3%) |
| **30–39** | 5,766 (19.3%) | 5,607 (22.2%) | 219 (10.7%) | 225 (12.6%) | 13 (2.9%) | 12 (3.6%) |
| **40–49** | 3,963 (13.3%) | 3,391 (13.4%) | 142 (7.0%) | 242 (13.6%) | 19 (4.3%) | 24 (7.3%) |
| **50–59** | 3,457 (11.6%) | 2,606 (10.3%) | 226 (11.1%) | 285 (16.0%) | 40 (9.0%) | 43 (13.0%) |
| **60–69** | 2,639 (8.8%) | 1,755 (6.9%) | 324 (15.9%) | 298 (16.7%) | 77 (17.3%) | 71 (21.5%) |
| **70–79** | 1,270 (4.3%) | 828 (3.3%) | 354 (17.3%) | 262 (14.7%) | 100 (22.4%) | 78 (23.6%) |
| **80–89** | 699 (2.3%) | 369 (1.5%) | 305 (14.9%) | 176 (9.9%) | 123 (27.6%) | 59 (17.8%) |
| **≥90** | 254 (0.9%) | 111 (0.4%) | 118 (5.8%) | 68 (3.8%) | 67 (15.0%) | 43 (13.0%) |
| **Unknown or missing** | 0 (0.0%) | 0 (0.0%) | 0 (0.0%) | 0 (0.0%) | 0 (0.0%) | 0 (0.0%) |
|  |  |  |  |  |  |  |
| **Race/ethnicity** |  |  |  |  |  |  |
| **Non-Hispanic Asian/Pacific Islander** | 3,648 (12.2%) | 2,048 (8.1%) | 168 (8.2%) | 118 (6.6%) | 43 (9.6%) | 20 (6.0%) |
| **Non-Hispanic Black/African American** | 4,816 (16.1%) | 5,014 (19.8%) | 690 (33.8%) | 592 (33.3%) | 138 (30.9%) | 111 (33.5%) |
| **Hispanic/Latino** | 7,653 (25.6%) | 5,967 (23.6%) | 490 (24.0%) | 466 (26.2%) | 96 (21.5%) | 77 (23.3%) |
| **Non-Hispanic White** | 6,712 (22.5%) | 7,280 (28.8%) | 430 (21.1%) | 432 (24.3%) | 142 (31.8%) | 102 (30.8%) |
| **Non-Hispanic Other** | 200 (0.7%) | 537 (2.1%) | 23 (1.1%) | 58 (3.3%) | 11 (2.5%) | 12 (3.6%) |
| **Unknown** | 6,837 (22.9%) | 4,426 (17.5%) | 241 (11.8%) | 114 (6.4%) | 16 (3.6%) | 9 (2.7%) |
|  |  |  |  |  |  |  |
| **Congregate setting resident[[1]](#footnote-2)** | | | | | | |
| **Yes** | 347 (1.2%) | 119 (0.5%) | 104 (5.1%) | 59 (3.3%) | 60 (13.5%) | 36 (10.9%) |
| **No** | 28,628 (95.9%) | 24,951 (98.7%) | 1,856 (90.9%) | 1,713 (96.2%) | 379 (85.0%) | 295 (89.1%) |
| **Unknown or missing** | 891 (3.0%) | 202 (0.8%) | 82 (4.0%) | 8 (0.4%) | 7 (1.6%) | 0 (0.0%) |
|  |  |  |  |  |  |  |
| **Census tract-based poverty level[[2]](#footnote-3)** | | | | | | |
| **Low** | 8,801 (29.5%) | 7,816 (30.9%) | 343 (16.8%) | 371 (20.8%) | 89 (20.0%) | 77 (23.3%) |
| **Medium** | 9,254 (31.0%) | 7,830 (31.0%) | 708 (34.7%) | 562 (31.6%) | 169 (37.9%) | 103 (31.1%) |
| **High** | 5,358 (17.9%) | 4,538 (18.0%) | 386 (18.9%) | 380 (21.3%) | 77 (17.3%) | 56 (16.9%) |
| **Very high** | 5,094 (17.1%) | 4,255 (16.8%) | 498 (24.4%) | 420 (23.6%) | 101 (22.6%) | 87 (26.3%) |
| **Unknown or missing** | 1,359 (4.6%) | 833 (3.3%) | 107 (5.2%) | 47 (2.6%) | 10 (2.2%) | 8 (2.4%) |
|  |  |  |  |  |  |  |
| **Prior COVID-19 diagnosis** | | | | | | |
| **Yes** | 2,137 (7.2%) | 1,251 (5.0%) | 130 (6.4%) | 24 (1.3%) | 19 (4.3%) | 3 (0.9%) |
| **No** | 27,729 (92.8%) | 24,021 (95.0%) | 1,912 (93.6%) | 1,756 (98.7%) | 427 (95.7%) | 328 (99.1%) |
|  |  |  |  |  |  |  |
| **Days since prior diagnosis** | | | | | | |
| **91–180** | 153 (7.2%) | 99 (7.9%) | 12 (9.2%) | 10 (41.7%) | 3 (15.8%) | 2 (66.7%) |
| **180–269** | 166 (7.8%) | 218 (17.4%) | 10 (7.7%) | 8 (33.3%) | 1 (5.3%) | 0 (0.0%) |
| **270–359** | 764 (35.8%) | 203 (16.2%) | 47 (36.2%) | 2 (8.3%) | 3 (15.8%) | 0 (0.0%) |
| **360–449** | 533 (24.9%) | 637 (50.9%) | 30 (23.1%) | 1 (4.2%) | 2 (10.5%) | 0 (0.0%) |
| **≥450** | 521 (24.4%) | 93 (7.4%) | 31 (23.8%) | 3 (12.5%) | 10 (52.6%) | 1 (33.3%) |
|  |  |  |  |  |  |  |
| **Number of COVID-19 vaccine doses** | | | | | | |
| **0** | 10,863 (36.4%) | 16,006 (63.3%) | 1,105 (54.1%) | 1,405 (78.9%) | 271 (60.8%) | 258 (77.9%) |
| **1** | 1,502 (5.0%) | 680 (2.7%) | 87 (4.3%) | 53 (3.0%) | 21 (4.7%) | 10 (3.0%) |
| **2** | 13,129 (44.0%) | 8,443 (33.4%) | 693 (33.9%) | 310 (17.4%) | 119 (26.7%) | 58 (17.5%) |
| **3** | 4,372 (14.6%) | 143 (0.6%) | 157 (7.7%) | 12 (0.7%) | 35 (7.8%) | 5 (1.5%) |
|  |  |  |  |  |  |  |
| **Days since 14 day**-period had passed **after most recent COVID-19 vaccine dose** | | | | | | |
| **<90** | 6,906 (36.3%) | 1,788 (19.3%) | 263 (28.1%) | 82 (21.9%) | 46 (26.3%) | 17 (23.3%) |
| **90–179** | 3,295 (17.3%) | 3,889 (42.0%) | 180 (19.2%) | 133 (35.5%) | 24 (13.7%) | 27 (37.0%) |
| **180–269** | 6,735 (35.4%) | 3,245 (35.0%) | 350 (37.4%) | 141 (37.6%) | 63 (36.0%) | 27 (37.0%) |
| **≥270** | 1,967 (10.4%) | 314 (3.4%) | 141 (15.0%) | 16 (4.3%) | 42 (24.0%) | 1 (1.4%) |

**Table S2. Relative risk for COVID-19 hospitalization among patients testing positive for SARS-CoV-2 infection during periods of Omicron compared with Delta predominance, overall and according to vaccination status, New York City, August 2021–January 2022.**

|  | **Hospitalized** | **Not hospitalized** | **Crude relative risk  (95% CI)** | **p value** | **Adjusted relative risk (95% CI)** | **p value** |
| --- | --- | --- | --- | --- | --- | --- |
| **Variant** |  |  |  |  |  |  |
| **Delta** | 8,268/158,799 (5.2%) | 150,531/158,799 (94.8%) | 1 (ref) |  | 1 (ref) |  |
| **Omicron** | 16,025/488,053 (3.3%) | 472,028/488,053 (96.7%) | 0.62 (0.55, 0.70) | <.001 | 0.72 (0.63, 0.82) | <.001 |
| **By vaccination status** |  |  |  |  |  |  |
| **None or only one dose** |  |  |  | <.001 |  | <.001 |
| **Delta** | 6,391/107,356 (6.0%) | 100,965/107,356 (94.0%) | 1 (ref) |  | 1 (ref) |  |
| **Omicron** | 8,837/218,185 (4.1%) | 209,348/218,185 (95.9%) | 0.68 (0.66, 0.70) | <.001 | 0.65 (0.63, 0.67) | <.001 |
| **Two doses** |  |  |  |  |  |  |
| **Delta** | 1,849/50,897 (3.6%) | 49,048/50,897 (96.4%) | 1 (ref) |  | 1 (ref) |  |
| **Omicron** | 5,558/200,284 (2.8%) | 194,726/200,284 (97.2%) | 0.76 (0.73, 0.80) | <.001 | 0.98 (0.93, 1.04) | 0.58 |
| **Three doses** |  |  |  |  |  |  |
| **Delta** | 28/546 (5.1%) | 518/546 (94.9%) | 1 (ref) |  | 1 (ref) |  |
| **Omicron** | 1,630/69,584 (2.3%) | 67,954/69,584 (97.7%) | 0.46 (0.32, 0.66) | <.001 | 0.58 (0.40, 0.84) | 0.004 |
| **Restricted to Omicron** |  |  |  |  |  |  |
| **None or only one dose** | 8,837/218,185 (4.1%) | 209,348/218,185 (95.9%) | 1 (ref) |  | 1 (ref) |  |
| **Two doses** | 5,558/200,284 (2.8%) | 194,726/200,284 (97.2%) | 0.69 (0.66, 0.71) | <.001 | 0.77 (0.72, 0.82) | <.001 |
| **Three doses** | 1,630/69,584 (2.3%) | 67,954/69,584 (97.7%) | 0.58 (0.55, 0.61) | <.001 | 0.37 (0.35, 0.40) | <.001 |

Patients with Delta infection and 0–1 doses as referent:

|  | **Not hospitalized** | | **Hospitalized** | | **Crude relative risk (95% CI)** | | **p value** | **Adjusted relative risk (95% CI)** | | **p value** |
| --- | --- | --- | --- | --- | --- | --- | --- | --- | --- | --- |
|  | Delta | Omicron | Delta | Omicron | Delta | Omicron |  | Delta | Omicron |  |
| **None or one dose** | 100,965/310,313 (32.5%) | 209,348/310,313 (67.5%) | 6,391/15,228 (42.0%) | 8,837/15,228 (58.0%) | 1 (ref) | 0.68 (0.66, 0.70) | <.001 | 1 (ref) | 0.65 (0.63, 0.67) | <.001 |
| **Two doses** | 49,048/243,774 (20.1%) | 194,726/243,774 (79.9%) | 1,849/7,407 (25.0%) | 5,558/7,407 (75.0%) | 0.61 (0.58, 0.64) | 0.47 (0.45, 0.48) | . | 0.51 (0.47, 0.55) | 0.50 (0.47, 0.54) | . |
| **Three doses** | 518/68,472 (0.8%) | 67,954/68,472 (99.2%) | 28/1,658 (1.7%) | 1,630/1,658 (98.3%) | 0.86 (0.60, 1.24) | 0.39 (0.37, 0.42) | . | 0.42 (0.29, 0.62) | 0.24 (0.23, 0.26) | . |

**Table S3. Relative risk for COVID-19 death among patients testing positive for SARS-CoV-2 infection during periods of Omicron compared with Delta predominance, overall and according to vaccination status, New York City, August 2021–January 2022.**

|  | **Death** | **No death** | **Crude relative risk (95% CI)** | **p value** | **Adjusted relative risk (95% CI)** | **p value** |
| --- | --- | --- | --- | --- | --- | --- |
| **Variant** |  |  |  |  |  |  |
| **Delta** | 1,196/158,799 (0.8%) | 157,603/158,799 (99.2%) | 1 (ref) |  | 1 (ref) |  |
| **Omicron** | 2,696/488,053 (0.6%) | 485,357/488,053 (99.4%) | 0.66 (0.49, 0.89) | .006 | 0.81 (0.58, 1.13) | .22 |
| **By vaccination status** |  |  |  |  |  |  |
| **None or only one dose** |  |  |  | .11 |  | <.001 |
| **Delta** | 890/107,356 (0.8%) | 106,466/107,356 (99.2%) | 1 (ref) |  | 1 (ref) |  |
| **Omicron** | 1,534/218,185 (0.7%) | 216,651/218,185 (99.3%) | 0.85 (0.78, 0.92) | <.001 | 0.68 (0.63, 0.74) | <.001 |
| **Two doses** |  |  |  |  |  |  |
| **Delta** | 301/50,897 (0.6%) | 50,596/50,897 (99.4%) | 1 (ref) |  | 1 (ref) |  |
| **Omicron** | 865/200,284 (0.4%) | 199,419/200,284 (99.6%) | 0.73 (0.64, 0.83) | <.001 | 1.09 (0.95, 1.25) | .22 |
| **Three doses** |  |  |  |  |  |  |
| **Delta** | 5/546 (0.9%) | 541/546 (99.1%) | 1 (ref) |  | 1 (ref) |  |
| **Omicron** | 297/69,584 (0.4%) | 69,287/69,584 (99.6%) | 0.47 (0.19, 1.12) | .09 | 0.72 (0.27, 1.93) | .52 |
| **Restricted to Omicron** |  |  |  |  |  |  |
| **None or only one dose** | 1,534/218,185 (0.7%) | 216,651/218,185 (99.3%) | 1 (ref) |  | 1 (ref) |  |
| **Two doses** | 865/200,284 (0.4%) | 199,419/200,284 (99.6%) | 0.61 (0.57, 0.67) | <.001 | 0.82 (0.69, 0.98) | .03 |
| **Three doses** | 297/69,584 (0.4%) | 69,287/69,584 (99.6%) | 0.61 (0.54, 0.69) | <.001 | 0.39 (0.31, 0.47) | <.001 |

Patients with Delta infection and 0–1 doses as referent:

|  | **No death** | | **Death** | | **Crude relative risk  (95% CI)** | | **p value** | **Adjusted relative risk (95% CI)** | | **p value** |
| --- | --- | --- | --- | --- | --- | --- | --- | --- | --- | --- |
|  | Delta | Omicron | Delta | Omicron | Delta | Omicron |  | Delta | Omicron |  |
| **None or one dose** | 106,466/323,117 (32.9%) | 216,651/323,117 (67.1%) | 890/2,424 (36.7%) | 1,534/2,424 (63.3%) | 1 (ref) | 0.85 (0.78, 0.92) | .11 | 1 (ref) | 0.68 (0.63, 0.74) | <.001 |
| **Two doses** | 50,596/250,015 (20.2%) | 199,419/250,015 (79.8%) | 301/1,166 (25.8%) | 865/1,166 (74.2%) | 0.71 (0.63, 0.81) | 0.52 (0.47, 0.57) | . | 0.51 (0.42, 0.63) | 0.56 (0.46, 0.68) | . |
| **Three doses** | 541/69,828 (0.8%) | 69,287/69,828 (99.2%) | 5/302 (1.7%) | 297/302 (98.3%) | 1.10 (0.46, 2.65) | 0.51 (0.45, 0.59) | . | 0.36 (0.13, 0.99) | 0.26 (0.21, 0.33) | . |

**Figure S1.** Eligibility for secondary analysis of New York City residents with SARS-CoV-2 Delta or Omicron variant infection based on whole-genome sequencing result.

1,278,809 New York City residents with laboratory-positive SARS-CoV-2 infections, July 2021–January 2022

Infection with Delta (N=26,376) or Omicron (N=31,233)

1,221,200 infections excluded. Exclusions not mutually exclusive:

1,218,627 with no whole-genome sequencing (WGS) result

467 with WGS result other than Delta or Omicron

2,044 with discordant WGS results

6 among persons with >1 diagnosis of Delta infection >90 days apart

80 among persons with both Delta and Omicron infections

2,471 excluded as vaccinated with Ad26.COV2 from Janssen (Johnson & Johnson)

Eligible study population: Infection with Delta (N = 25,272) or Omicron (N = 29,866) and unvaccinated or vaccinated with a COVID-19 mRNA vaccine

**Infection with hospitalization**:

Yes: Delta (N = 1,780), Omicron (N = 2,042)

No: Delta (N = 23,492), Omicron (N=27,824)

**Infection with hospitalization and ED presentation with COVID-19–like illness**:

Yes: Delta (N = 501), Omicron (N = 295)

No: Delta (N = 23,492), Omicron (N = 27,824)

Infection with delta (N=#) or Omicron (N=#) with hospitalization and emergency department presentation with COVID-19-like-illness

3,026 hospitalized and excluded:

745 did not present to ED within +/- 14 days of diagnosis

2,281 presented to ED without COVID-19–like illness

**Infection with death**:

Yes: Delta (N = 331), Omicron (N = 446)

No: Delta (N = 24,941), Omicron (N = 29,420)

**Infection with death and COVID-19 on death certificate**:

Yes: Delta (N = 295), Omicron (N = 348)

No: Delta (N = 24,941), Omicron (N = 29,420)

Infection with delta (N=#) or Omicron (N=#) with hospitalization and emergency department presentation with COVID-19-like-illness

134 deaths excluded because COVID-19 not listed as a cause on death certificate

**Figure S2. Relative risk (RR) for (A) hospitalization with COVID-19–like illness presentation and (B) death with COVID-19 indicated on the death certificate, among patients testing positive for SARS-CoV-2 infection during periods of Omicron compared with Delta predominance overall and according to vaccination status, New York City, August 2021–January 2022.**

(A) Hospitalization with COVID-19–like illness presentation (B) Death with COVID-19 indicated on the death certificate


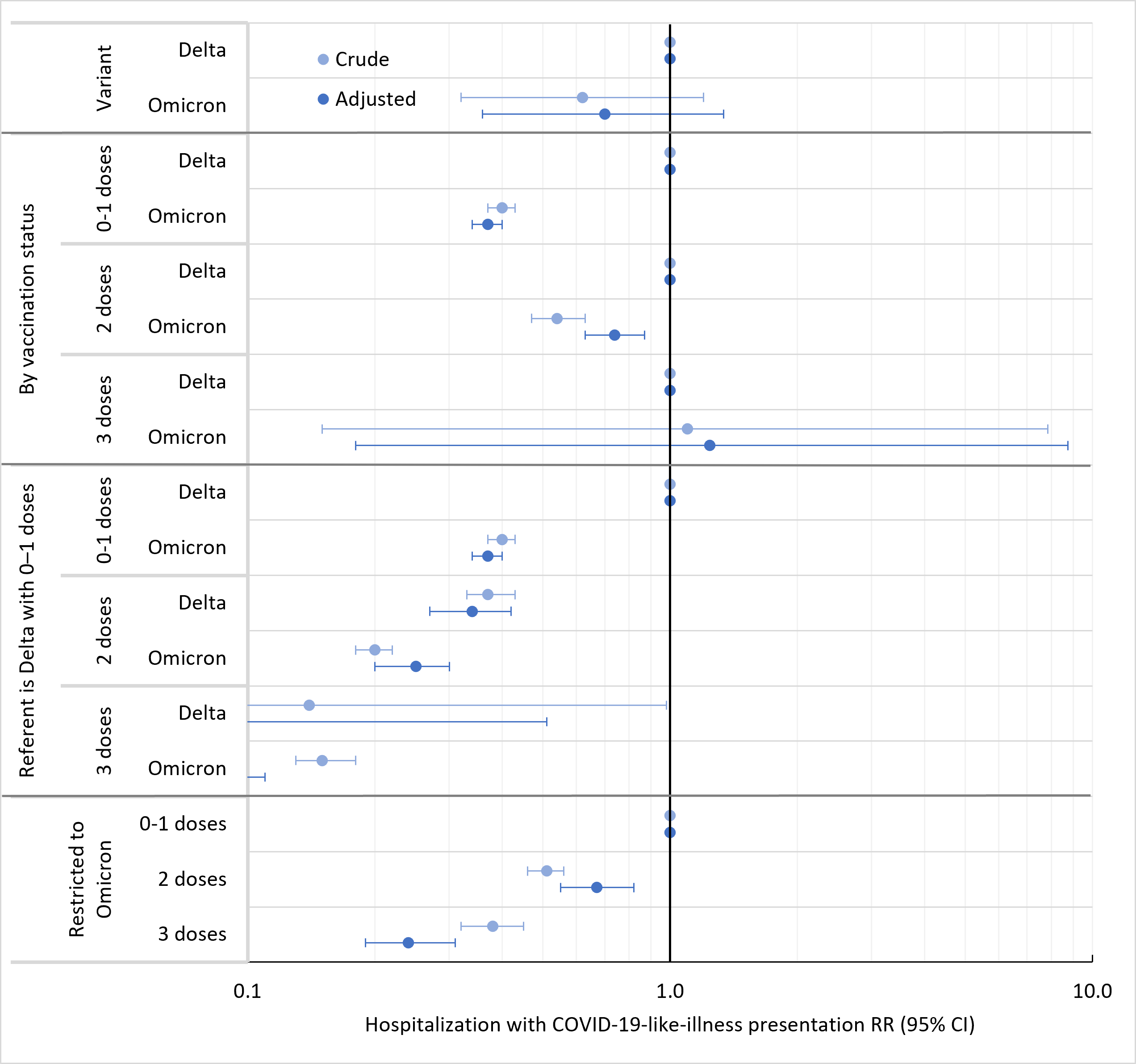

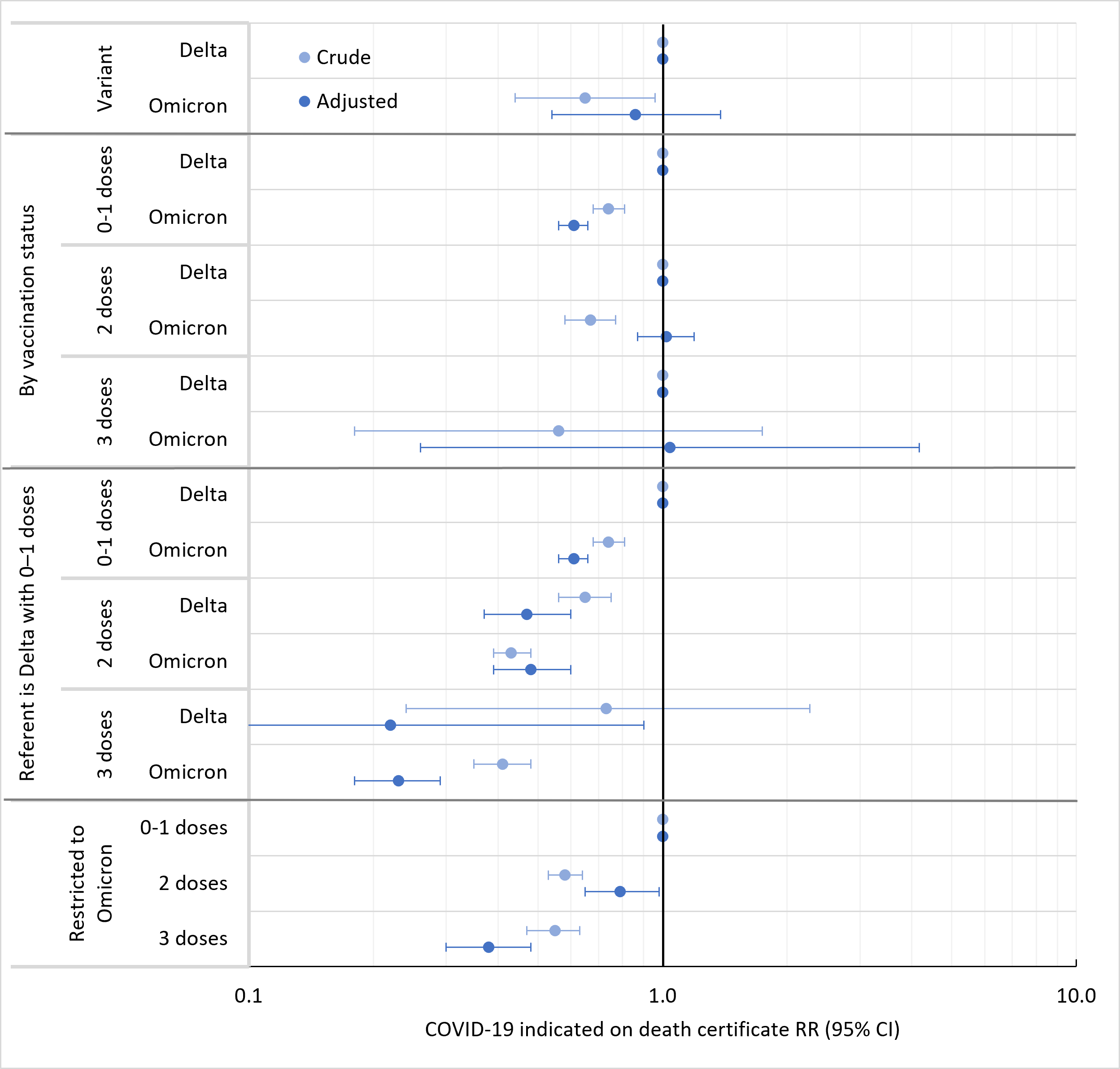


**Figure S3. Relative risk (RR) for COVID-19 hospitalization and death among patients with Omicron compared with Delta sequencing results, overall and according to vaccination status, New York City, July 2021–January 2022.**

(A) Hospitalization (B) Death


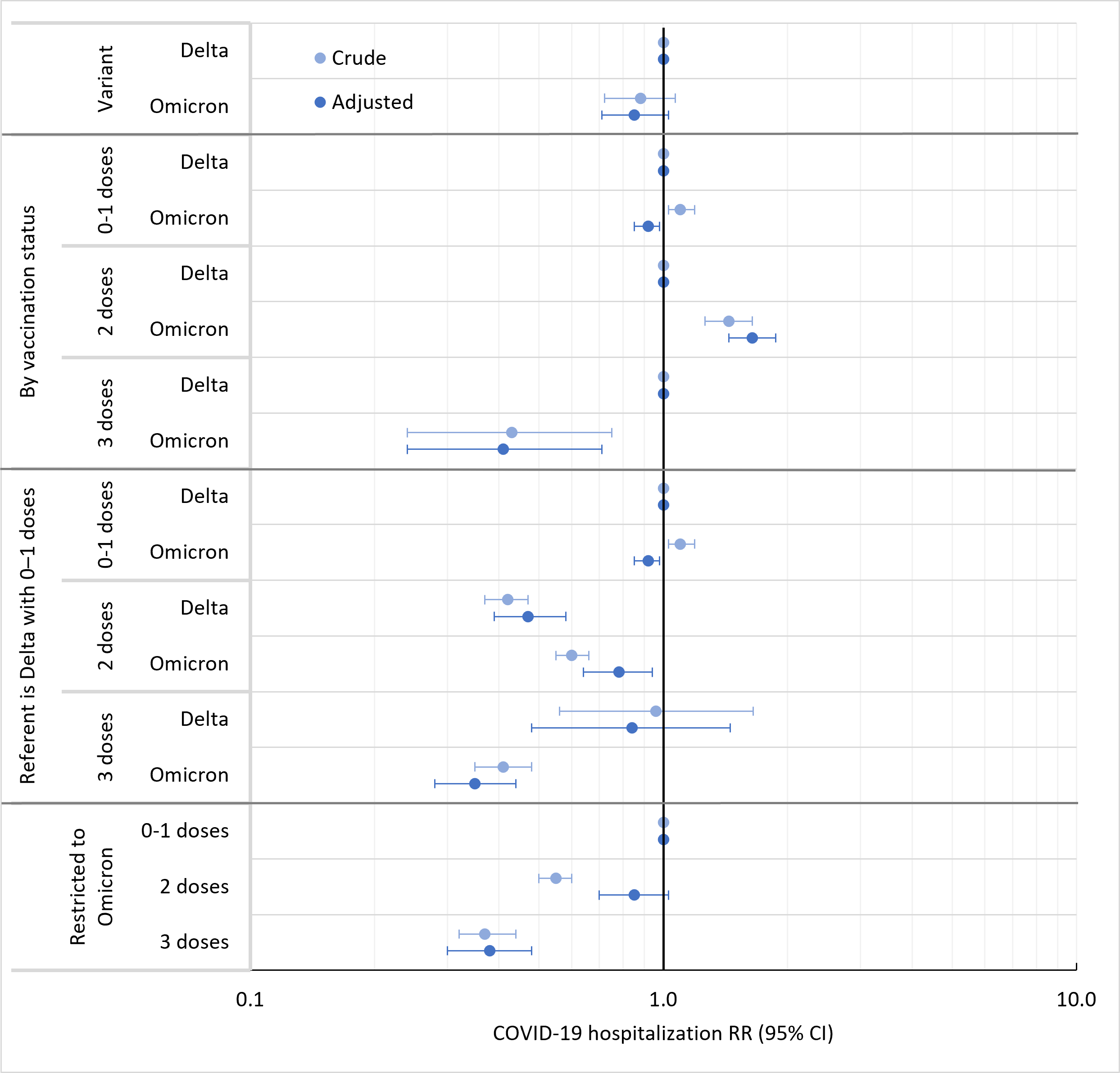

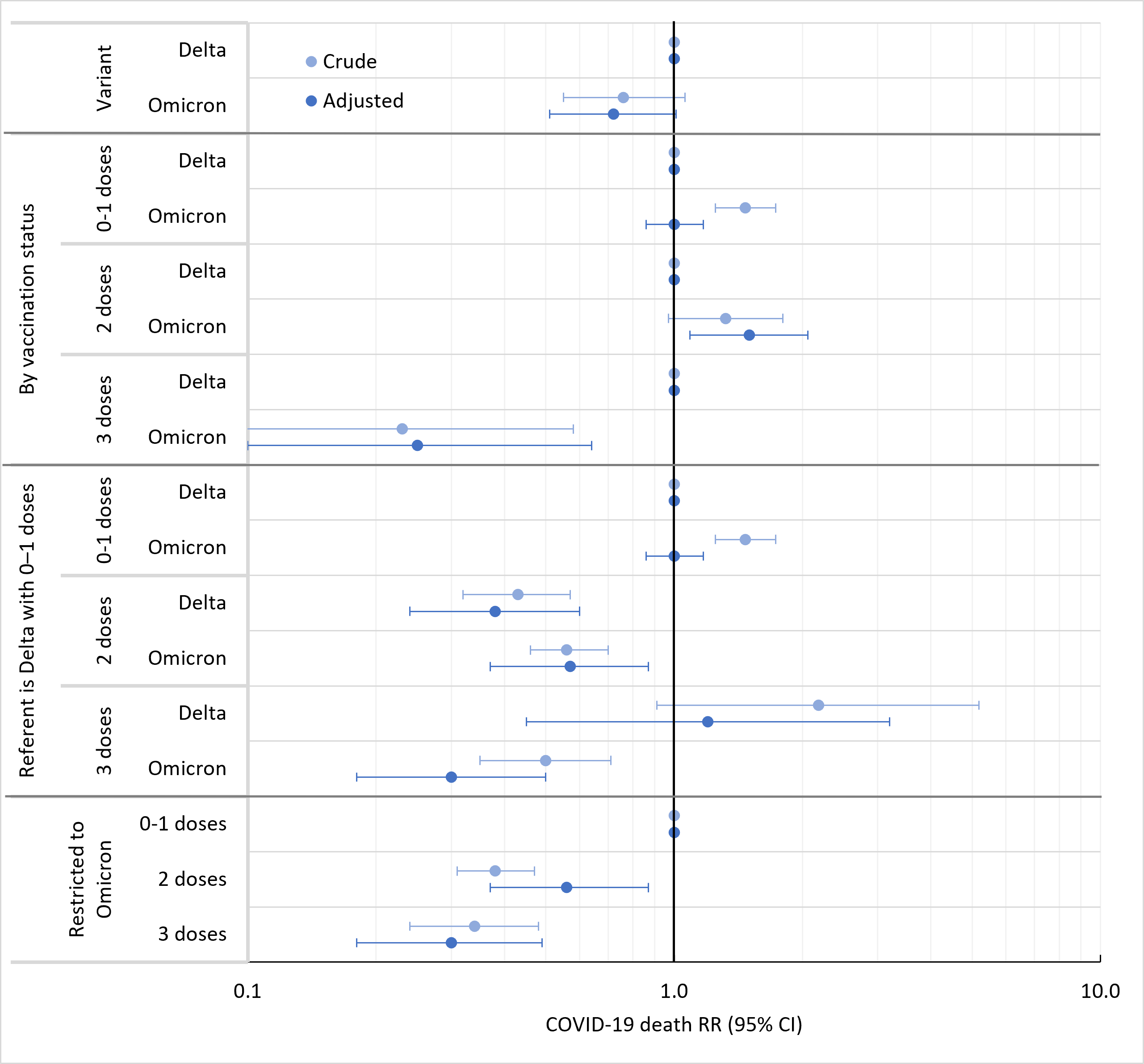


1. Congregate settings defined as having a residential address as of diagnosis of a nursing home, jail, or prison. [↑](#footnote-ref-2)
2. Low poverty defined as <10% of residents below the federal poverty level, medium as 10% to <20%, high as 20 to <30%, and very high as ≥30%. [↑](#footnote-ref-3)
